# Hypertrophic cardiomyopathy: a heart in need of an energy bar?

**DOI:** 10.3389/fphys.2014.00309

**Published:** 2014-08-19

**Authors:** Styliani Vakrou, M. Roselle Abraham

**Affiliations:** Division of Cardiology, School of Medicine, Johns Hopkins UniversityBaltimore, MD, USA

**Keywords:** hypertrophic cardiomyopathy, mitochondria, calcium handling, bioenergetic deficit, induced pluripotent stem cells (iPSCs)

## Abstract

Hypertrophic cardiomyopathy (HCM) has been recently recognized as the most common inherited cardiovascular disorder, affecting 1 in 500 adults worldwide. HCM is characterized by myocyte hypertrophy resulting in thickening of the ventricular wall, myocyte disarray, interstitial and/or replacement fibrosis, decreased ventricular cavity volume and diastolic dysfunction. HCM is also the most common cause of sudden death in the young. A large proportion of patients diagnosed with HCM have mutations in sarcomeric proteins. However, it is unclear how these mutations lead to the cardiac phenotype, which is variable even in patients carrying the same causal mutation. Abnormalities in calcium cycling, oxidative stress, mitochondrial dysfunction and energetic deficiency have been described constituting the basis of therapies in experimental models of HCM and HCM patients. This review focuses on evidence supporting the role of cellular metabolism and mitochondria in HCM.

## Clinical features of hypertrophic cardiomyopathy

Hypertrophic cardiomyopathy (HCM) was first recognized as a clinical entity, approximately 55 years ago (Brock, 1957; Teare, 1958; Cohen et al., 1964; Ross et al., 1966). It is the most common inherited cardiac disease with an estimated prevalence of 1: 500 in young individuals (Maron, 2002). Inheritance is autosomal dominant, with variable penetrance in 50–60% of patients; causal mutations have not been identified in 40–50% of HCM patients (Jarcho et al., 1989; Solomon et al., 1990; Marian and Roberts, 2001; Marian, 2002). Nine different chromosomal loci have been linked to HCM with the majority of genes encoding cardiac sarcomeric proteins (Jarcho et al., 1989; Geisterfer-Lowrance et al., 1990; Watkins et al., 1993; Thierfelder et al., 1994). The most common mutations occur in genes encoding for β-myosin heavy chain (35%), myosin binding protein C (20%), troponin T (5%) and α-tropomyosin (<3%), which have roles in cardiac excitation-contraction coupling (Maron and Maron, 2013).

**Figure 1:**
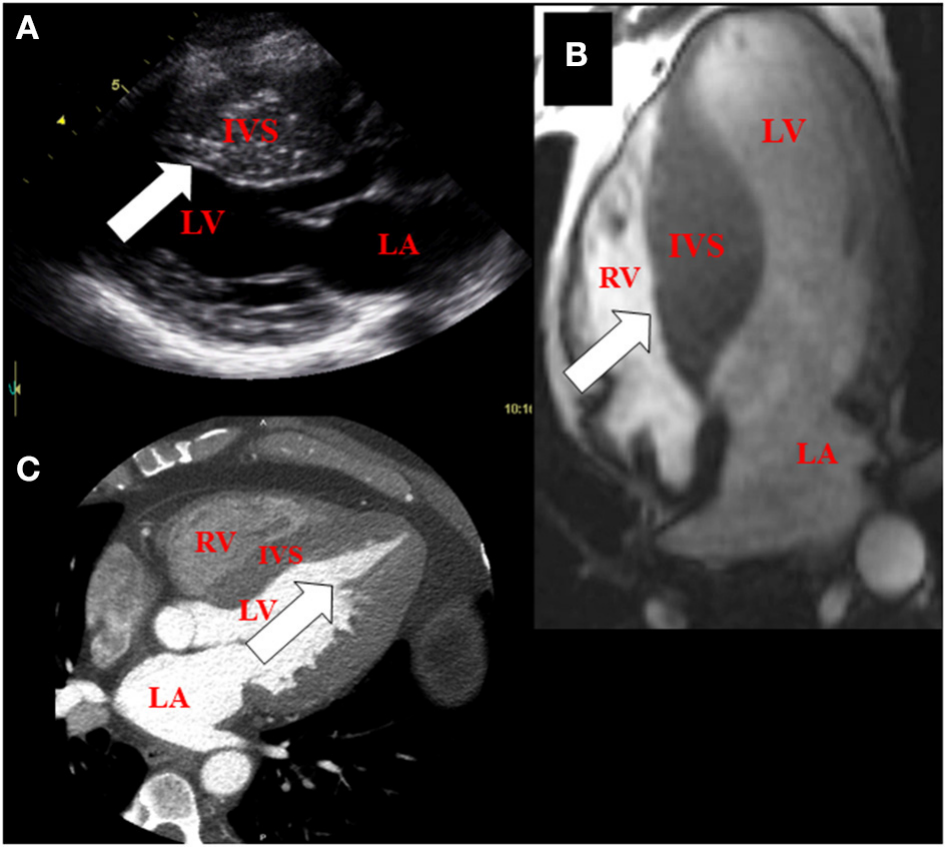
**Imaging features of hypertrophic cardiomyopathy using multi-modality cardiac imaging in patients. (A)** Basal hypertrophy of the inter-ventricular septum (arrow) using echocardiography (parasternal long axis view of the heart). **(B)** Mid-septal hypertrophy (arrow) using magnetic resonance imaging (4-chamber view of the heart). **(C)** Apical hypertrophy (arrow) using Computed tomography (4-chamber view of the heart). LA, left atrium; IVS, inter-ventricular septum; LV, left ventricle; RV, right ventricle.

Asymmetric ventricular hypertrophy and left ventricular outflow tract obstruction with normal or hyperdynamic systolic function are common morphologic manifestations of HCM (Maron et al., 2003). However, clinical phenotype is variable even among individuals carrying the same causal mutation due to effects of modifier genes, which are largely unknown (Seidman and Seidman, 2001). As a result degree and location (mid-ventricular, septal, apical and concentric) of hypertrophy and obstruction are variable in patients with HCM (Figure 1). Clinical presentation is also heterogeneous, spanning the spectrum from individuals who are largely asymptomatic, to patients with moderate to severe symptoms, ranging from angina, exercise intolerance to heart failure (requiring heart transplantation), atrial fibrillation and sudden cardiac death (Maron, 2002; Maron et al., 2002; Gersh et al., 2011). Irrespective of the causal mutation, pathologically, HCM is characterized by myocyte hypertrophy, myocyte disarray and fibrosis (Ho et al., 2010).

Pioneering work by several groups has revealed the molecular genetics and biophysical mechanisms underlying HCM. A variety of functional defects, including altered Ca^2+^ sensitivity and/or affinity, myofibrillar ATPase activity, Ca^2+^ handling, cross-bridge dynamics, impaired energetics, oxidative stress and electrophysiologic abnormalities have been identified in experimental models (Straceski et al., 1994; Spindler et al., 1998; Blanchard et al., 1999; Gao et al., 1999; Georgakopoulos et al., 1999; Tardiff et al., 1999; Solaro et al., 2002; Javadpour et al., 2003; Adhikari et al., 2004; Szczesna-Cordary et al., 2004; Ertz-Berger et al., 2005; Hernandez et al., 2005; Robinson et al., 2007; Greenberg et al., 2009, 2010; Guinto et al., 2009; Mettikolla et al., 2011; Puglisi et al., 2014) and patients (Haq et al., 2001; Crilley et al., 2003; Nakamura et al., 2005; Dimitrow et al., 2009; Unno et al., 2009; Ho et al., 2010; Bravo et al., 2012; Coppini et al., 2013; Lin et al., 2013; Gruner et al., 2014). Since HCM-causing mutations increase the energetic cost of tension development, it has been hypothesized that excessive sarcomeric energy use leads to the HCM phenotype (Blair et al., 2001; Crilley et al., 2003; Abozguia et al., 2010). We (Abraham et al., 2013) and others (Jung et al., 1998, 2000; Crilley et al., 2003; Timmer et al., 2011) have shown reduced PCr/ATP ratios in HCM patients with both established left ventricular hypertrophy and in the pre-hypertrophic stage, which suggests that bioenergetic deficits may be a primary cause of myocardial remodeling.

## Evidence of HCM as a metabolic disease

^31^P NMR spectroscopy studies have demonstrated a reduction in ATP reserve in HCM mouse models following inotropic stimulation (Spindler et al., 1998; Javadpour et al., 2003). Evidence for energy deficit in HCM has also been obtained from patient studies revealing increased glucose uptake (Tadamura et al., 1996), reduction of PCr/ATP ratios in pre-hypertrophic patients (Crilley et al., 2003) and reduced coronary sinus pH despite non-limiting capillary oxygen pressures (possibly indicating up-regulation of glycolysis with lactate generation) (Tadamura et al., 1996; Jung et al., 1998; Ashrafian et al., 2003; Keren et al., 2008). However, it is not known whether the energy deficit paradigm can be generalized to *all* HCM patients, at *all* stages of the disease. Furthermore, the molecular basis of the energetic deficits in HCM and their attendant consequences has been understudied.

**Figure 2:**
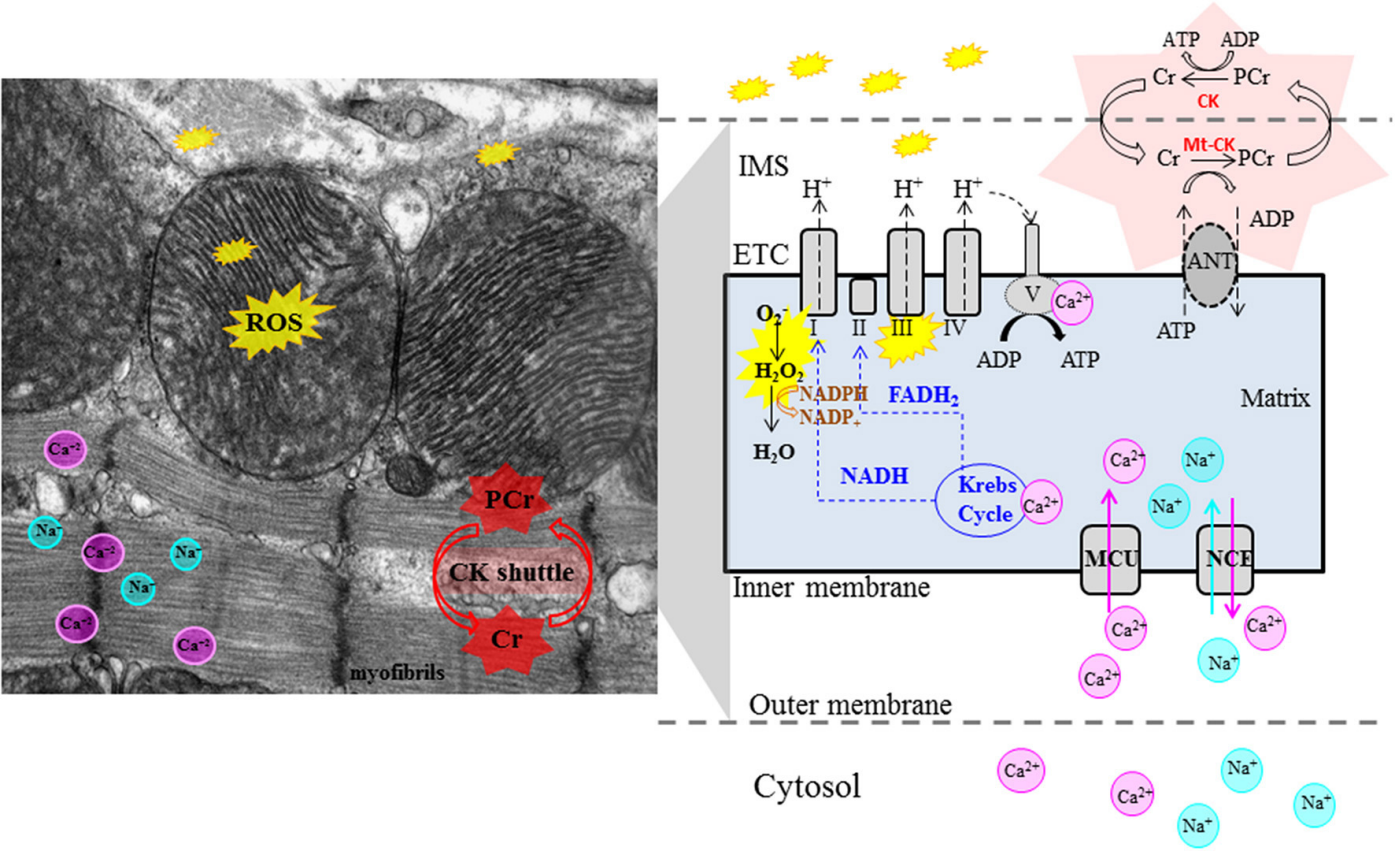
**Role of Mitochondria in pathogenesis of cardiac phenotype in HCM.** Left panel: electron microscopy image of mouse heart. Right panel: schematic illustrating mitochondrial physiology. The Krebs cycle generates reducing equivalents (NADH, FADH_2_) that drive proton pumping, establish the proton-motive force across the mitochondrial inner membrane and contribute to ROS scavenging. Mitochondrial ATP synthase (complex V) couples proton influx to ATP generation. Matrix concentrations of Ca^2+^ and Na^+^ play an important role in control of oxidative phosphorylation. Mitochondria are the main source of ATP generation and important source of ROS (from complexes I and III) in cardiac myocytes. Abnormalities in mitochondrial function, reduced CK flux, oxidative stress and impaired Ca^2+^ handling have been implicated in generation of the cardiac phenotype in HCM. Cr, creatine; PCr, creatine-phosphate, Mt-CK, mitochondrial creatine kinase; ANT, Adenine nucleotide translocator; ETC, electron transport chain; IMS, inter-membrane space; MCU, mitochondrial calcium uniporter; NCE, mitochondrial Na^+^−Ca^2+^ exchanger.

In the heart, ATP supply is tightly regulated to meet energetic demands of the myofilaments. The mechanisms by which cardiac energetics is finely tuned are still a matter of considerable debate, but there is emerging consensus on the importance of two regulators, Ca^2+^ and ADP (Cortassa et al., 2006; Saks et al., 2006; Balaban, 2009). During contraction, Ca^2+^-induced Ca^2+^ release from the sarcoplasmic reticulum floods the cytoplasm where it binds the thin filament regulatory protein Troponin C, thereby initiating contraction (Bers, 2002). Coordinate activation of ATP production arises because mitochondria, positioned close to the SR, take up Ca^2+^ via the mitochondrial calcium uniporter (MCU) (Maack and O'Rourke, 2007). Mitochondrial matrix calcium regulates 3 key enzymes in the tricarboxylic acid (TCA) cycle that harnesses the decarboxylation of acetyl-CoA to yield reduced nicotinamide adenine dinucleotide (NADH) which fuels the respiratory electron transport chain (ETC) and is converted to NADPH which plays a critical role in maintaining mitochondrial anti-oxidant capacity (McCormack and Denton, 1990; Hansford and Zorov, 1998; Liu et al., 2014); Mitochondrial Ca^2+^ can also directly stimulate respiratory complex activity, including the mitochondrial ATP synthase (*F1F0* ATPase) (Territo et al., 2000). Thus, Ca^2+^ coordinately regulates ATP-consuming myofilaments and ATP-generating oxidative phosphorylation (Figure 2).

Ca^2+^ uptake by mitochondria is dependent on cytosolic Na^+^ levels, which has been demonstrated to be elevated in experimental models of heart failure (Liu and O'Rourke, 2008) and failing human hearts (Pieske and Houser, 2003). The O'Rourke group has demonstrated that elevated cytosolic Na^+^ increases the rate of the mitochondrial Na^+^−Ca^2+^ exchanger (mNCE), which promotes mitochondrial Ca^2+^ efflux and decreases the mitochondria's ability to accumulate Ca^2+^ during conditions of high demand (Maack et al., 2006; Liu and O'Rourke, 2013). Without Ca^2+^-induced Kreb's cycle stimulation, NADH and NADPH become more oxidized and are unable to recharge antioxidant systems, leading to ROS accumulation in the mitochondrial matrix and release into the cytosol (Kohlhaas et al., 2010; Gauthier et al., 2013; Liu and O'Rourke, 2013). Partial inhibition of mNCE by CGP-37157 attenuated adverse ventricular remodeling and was anti-arrhythmic in a guinea pig model of pressure overload (Liu et al., 2014). A recent study of Ranolazine, an inhibitor of late Na^+^ current, that is known to contribute to cytosolic Na^+^ overload revealed salutary effects on action potential duration and arrhythmias in cardiac myocytes of HCM patients who underwent myectomy (Coppini et al., 2013). Investigation of cytosolic Na^+^ levels (Gao et al., 2013) and mitochondrial Ca^2+^ handling is needed in order to assess whether altered mitochondrial Ca^2+^ dynamics contribute to energetic deficits and oxidative stress in HCM (Nakamura et al., 2005; Senthil et al., 2005; Marian et al., 2006; Dimitrow et al., 2009).

Conditions such as exercise that quickly elevate heart rate impose energetic demands that can quickly exceed Ca^2+^-regulated supply. In these cases, the by-product of myofilament ATPase activity, ADP, provides vital feedback stimulation of energy in two ways. Firstly the original work by Britton Chance and colleagues showed that the rate of ATP formation by Complex V is driven by the concentration of ADP. Myofilaments also possess a local ATP-buffering capacity maintained by cytosolic creatine kinase (CK). In high work conditions, ADP accumulation is sensed by CK, which catalyzes phosphoryl group transfer from phosphocreatine to regenerate ATP (Saks et al., 2006; Balaban, 2009) (Figure 2). Our studies in patients from a family carrying the R403Q mutation in myosin heavy chain (MHC) revealed a 43% reduction in forward CK flux at rest, indicating reduced metabolic reserve (Abraham et al., 2013). A recent study by Critoph et al. revealed reduced cardiac reserve secondary to blunted increase in cardiac output, in HCM patients undergoing exercise stress testing (Critoph et al., 2014). NMR studies in mice with R403Q-MHC (Spindler et al., 1998) and R92-TNT (Javadpour et al., 2003; He et al., 2007) mutations verified impaired myocardial energetics during inotropic stimulation. The decreased [PCr], increased [Pi], [ADP] and unchanged or decreased [ATP] can result in reduction in the calculated free energy release from ATP hydrolysis (lΔGl) (Spindler et al., 1998) which in turn can impair the function of cellular ATPases (e.g., myosin ATPase, Na^+^−K^+^-ATPase) and Ca^2+^ pumps like SERCA, leading to systolic and/or diastolic dysfunction, reduction or blunted increase in stroke volume, increased levels of cytosolic Na^+^, Ca^2+^ and arrhythmias under conditions of high work load, such as intense exercise (Unno et al., 2009; Ashrafian et al., 2011; Watkins et al., 2011).

## The role of mitochondria in HCM: what we know

The vital role of mitochondria as providers of energy for the high demands of cardiac contractility is well recognized, as is their contribution to necrotic and apoptotic cell death (Seddon et al., 2007). More, recently, the novel role of mitochondria as signaling organelles has emerged, primarily through their ability to produce reactive oxygen species (ROS) -including superoxide (O^−^_2_), hydrogen peroxide (H_2_O_2_) and hydroxyl radicals (^.^OH)- and reactive nitrogen species, including nitric oxide (NO) and peroxynitrite (ONOO^−^) (Balaban et al., 2005; Figueira et al., 2013). ROS are best known for the damage they cause by directly oxidizing proteins, lipids, and DNA, but recent evidence suggests that the controlled and carefully modulated release of ROS from the mitochondrial network can activate specific signaling pathways or mediate reversible post-translational modifications of target proteins with pronounced effects on function (Terentyev et al., 2008; Bayeva and Ardehali, 2010). Additionally, because mitochondria are major determinants of the redox potential of both the pyrimidine nucleotide (NADH, NADPH) and thiol (GSH, thioredoxin) pools, they are important regulators of myocyte function (Stanley et al., 2011; Kembro et al., 2013; Liu and O'Rourke, 2013; Liu et al., 2014). However, very little is known about myocyte and mitochondrial redox potential in HCM (Figure 2).

Impairment of mitochondrial function and morphological disorganization has been reported in mouse models (Tardiff et al., 1999; Lucas et al., 2003) and in HCM patients (Unno et al., 2009). However, a systematic study of mitochondrial function is lacking. It is also unclear whether mitochondrial abnormalities are a primary event or secondary event in HCM. Of note, patients with mutations in mitochondrial DNA (Obayashi et al., 1992; Rotig et al., 1997; Okajima et al., 1998; Elliott and McKenna, 2004) can have a similar cardiac phenotype as HCM patients with sarcomeric protein mutations, suggesting that energetic deficits can lead to the cardiac phenotype of HCM.

The normal heart relies primarily on fatty acid oxidation for ATP generation (Abozguia et al., 2006; Ingwall, 2009). Pathologic hypertrophy is known to be associated with a reduction in fatty acid oxidation and increased reliance on glucose for ATP generation (Abozguia et al., 2006; Coppini et al., 2013). Positron emission tomography (PET) using ^11^C-acetate and 18FDG have been employed to study glucose and fatty acid oxidation in HCM patients (Grover-McKay et al., 1989; Nienaber et al., 1993; Perrone-Filardi et al., 1993; Tadamura et al., 1996; Tuunanen et al., 2007): some studies have found decrease/no change or increase in glucose uptake and the same is true for fatty acid oxidation, in hypertrophied and non-hypertrophied walls, when compared to controls. This may be attributable to differences in clinical characteristics (stage of disease, degree of hypertrophy, presence of microvascular dysfunction) and mutation status of the small number of patients who were studied. Since enzymes for fatty acid oxidation are located in mitochondria, it is unclear whether abnormalities in fatty acid oxidation are secondary to mitochondrial dysfunction. Metabolomic studies (Mayr, 2008) are needed in mouse models and HCM patients to obtain insights into metabolic remodeling and its role in generation of the cardiac phenotype in HCM.

## Arrhythmias in HCM—do mitochondria play a role?

Sudden cardiac death is the most dreaded and tragic phenotype, as it is often the first manifestation of the disease and occurs in asymptomatic and apparently healthy young individuals (Maron and Maron, 2013). The enhanced ventricular arrhythmogenicity has been attributed to abnormal cardiomyocyte orientation and alignment (disarray), microvascular ischemia, and fibrosis (Coppini et al., 2013).

Sarcomeres are known to sequester Ca^2+^ (bound: free ratio is 100:1). It has been hypothesized that HCM mutations may increase “Ca^2+^ trapping,” and through altered on–off kinetics may lead to altered Ca^2+^ signaling and arrhythmogenesis (Semsarian et al., 2002; Ashrafian et al., 2011).

Another possibility that has not been explored is energetic deficits, because energy compromise would be most marked when the heart is subject to increased work load, as is the case during high intensity exercise. In fact, e*xercise-induced* arrhythmias are common causes of sudden death and defibrillator discharges in HCM patients (Ommen and Gersh, 2009; Spirito et al., 2014). Since HCM is associated with high sarcomeric ATP consumption at rest, the ability of the heart to provide sufficient ATP for myosin ATPase, SERCA and membrane ATPases could be compromised during exercise, leading to cytosolic Na^+^ and Ca^2+^ overload and triggered activity leading to clinical arrhythmias (Watkins et al., 2011). Another possibility is the “*metabolic sink hypothesis*,” proposed by the O'Rourke group (Akar et al., 2005), wherein regional oxidative stress (ROS-induced ROS release) in mitochondria results in mitochondrial membrane depolarization, K_ATP−_channel opening and *reentrant arrhythmias* (Zorov et al., 2000, 2006; Aon et al., 2003, 2006; O'Rourke et al., 2005; Zhou et al., 2009; Cortassa et al., 2014).

## Therapies in HCM—all left ventricular hypertrophy is not created equal

There is a need for therapies that prevent/reverse the cardiac phenotype in HCM (Force et al., 2010). Drugs such as beta-adrenergic antagonists (e.g., Metoprolol), L-type Ca^2+^ channel blockers (e.g., Diltiazem, Verapamil) (Semsarian et al., 2002; Elliott and McKenna, 2004; Spirito and Autore, 2006), angiotensin II receptor antagonists (e.g., Losartan), (Lim et al., 2001; Lombardi et al., 2009; Shimada et al., 2013) carnitine palmitoyltransferase-1/2 inhibitor (Perhexiline) (Abozguia et al., 2010), antiarrhythmics (e.g., Disopyramide, Amiodarone), surgical myectomy and alcohol septal ablation (Sorajja et al., 2012) have been used to treat symptomatic HCM (Gersh et al., 2011). Antioxidant therapy with L-NAC was shown to prevent hypertrophy and fibrosis in experimental models of HCM (Marian et al., 2006) and is now in clinical trials (HALT-HCM study). Recently, there has been interest in the use of Ranolazine, based on beneficial effects on action potential duration and arrhythmias, in cardiac myocytes derived from HCM patients undergoing myectomy that exhibited evidence of electrophysiologic remodeling (increased late Na^+^ and Ca^2+^ currents, reduced repolarizing K^+^ currents) (Coppini et al., 2013). Two studies are currently under way to test the efficacy of ranolazine on exercise tolerance and diastolic function in symptomatic HCM patients (RESTYLE-HCM, Germany, Menarini) and to treat chest pain or dyspnea in patients with HCM (RHYME, USA) (Spoladore et al., 2012). However, it is not known whether cytosolic Na^+^ is increased early in the course of the disease (pre-hypertrophic stage) and whether it leads to mitochondrial dysfunction in any/all HCM mutations, or whether high levels of cytosolic Na^+^ occur after the onset of myocyte hypertrophy and/or symptoms. Most importantly, *none of the agents tested clinically have been demonstrated to change disease course in symptomatic patients* (Nagueh et al., 2010). Possible reasons may be that the pathophysiology of myocyte hypertrophy is mutation-specific and the extent of hypertrophy (a common clinical endpoint) is only one determinant of prognosis. Another possibility is that all HCM is not created equal and consequently, *individualized, mutation-specific therapies* need to be developed.

Hypertrophy is a compensatory response to myocardial injury. While hypertension and HCM can both cause left ventricular hypertrophy which may be indistinguishable by clinical imaging, the molecular mechanisms underlying myocyte hypertrophy are probably different based on an early study of cyclosporine, which prevented left ventricular hypertrophy in the TAC (transverse aortic constriction) model (that simulates increased afterload caused by hypertension) (Sussman et al., 1998), but expedited hypertrophy in HCM mice with a mutation (R403Q) in the α-MHC gene (Teekakirikul et al., 2010). Hence there is *need for further investigations to clarify the mechanisms underlying the cardiac phenotype in HCM in order to spur development of new therapeutic strategies and pre-clinical screening tests*.

## Future directions

Identification of mutations has defined the genetic causes of HCM in 50–60% of HCM patients, but the molecular mechanisms underlying myocyte hypertrophy, fibrosis and ventricular arrhythmias have not been completely elucidated (Force et al., 2010). It is unclear to what extent genetic variants of HCM exhibit a common mechanism of pathogenesis and to what extent they differ. It is also unknown why certain sarcomeric mutations are well tolerated while others are particularly pernicious in patients, but not in animal models.

Based on positive results in animal models, clinical trials have investigated Ca^2+^ channel blockers and inhibitors of the renin-angiotensin-aldosterone system in the HCM population, with limited success -possible reasons include differences in disease pathophysiology between HCM-causing mutations and differences in physiology between mouse and human myocytes. Hence, studies in human myocytes are needed to confirm results obtained in mouse models and develop therapies that modify the clinical course of disease. Since human heart tissue can only be obtained by heart biopsy or during surgery, it has been difficult to conduct human studies of disease pathophysiology in large numbers of HCM patients at various stages of disease.

Advances in IPSC (induced pluripotent stem cell) technology permit derivation of *human* cardiac myocytes obtained by differentiation of human IPSCs derived from HCM patients (Matsa et al., 2014). A recent study by the Wu group at Stanford demonstrated that myocytes differentiated from IPSCs (IPSC-CMs) recapitulate the HCM disease phenotype and can serve as a platform to test therapies (Lan et al., 2013). Furthermore, mitochondrial dysfunction resulting from low levels of Frataxin was also reproduced in IPSC-CMs derived from patients with Friedrich's ataxia (Hick et al., 2013). Hence, IPSC-CMs derived from HCM patients could serve as human model systems of HCM to investigate mitochondrial function and molecular mechanisms underlying cardiac phenotype, develop individualized screening tests and drug therapies in HCM patients with *known and unknown causal mutations*.

## Concluding remarks

HCM is caused by mutations in sarcomeric proteins in 50–60% of patients. These mutations have been shown to increase the energetic cost of tension development. However, it is unclear whether energetic deficits are involved in generation of the cardiac phenotype in *all* HCM patients and whether mitochondrial dysfunction precedes development of energetic deficits. Further investigation of mitochondrial function, metabolism and its relationship to cardiac function and electrophysiology in animal models of HCM and/or patient-derived myocytes is needed to clarify the molecular mechanisms underlying the cardiac phenotype in HCM and to design therapies that prevent, arrest and reverse the disease phenotype.

### Conflict of interest statement

The authors declare that the research was conducted in the absence of any commercial or financial relationships that could be construed as a potential conflict of interest.
